# The Role of Suction in Ureteroscopy: A Narrative Review

**DOI:** 10.7759/cureus.98074

**Published:** 2025-11-29

**Authors:** Vyshnavi Sathish

**Affiliations:** 1 Urology, Peninsula Deanery, Torbay Hospital, Torquay, GBR

**Keywords:** kidney stones, suction, ureterorenoscopy, ureteroscopy, urs

## Abstract

Kidney stone disease is a prevalent urological condition, affecting at least one in 10 individuals. The rising incidence of urolithiasis represents a significant burden on the National Health Service (NHS) and has driven major advances in endourology, including the use of flexible ureteroscopes (F-URS), high-efficiency laser fibres and techniques. Despite these advances, retrograde intrarenal surgery (RIRS) still has complications such as residual fragments and infection risk. Whilst suction technology has been utilised in percutaneous nephrolithotomy (PCNL) for over 25 years, its use in RIRS is new. This narrative review evaluates the safety and clinical efficacy of suction-assisted ureteroscopy (URS) in the management of kidney stones. A literature search was performed on PubMed and Google Scholar for studies published between January 2015 and October 2025, using the keywords ‘suction’, ‘ureteroscopy’, ‘URS’, ‘F-URS’, ‘kidney stones’, ‘FANS,’ ‘flexible and navigation suction sheaths’, ‘suction urethral access sheaths’, ‘SUAS’, ‘direct-in-scope suction’, ‘DISS’ and ‘ureterorenoscopy’. Suction-assisted URS reduces intrarenal pressure and operative time and improves visibility. Suction urethral access sheaths (SUAS) enhance stone clearance rate (stone-free rate (SFR)) and reduce post-operative complications. Flexible and navigation suction sheaths (FANS) integrate a continuous suction mechanism with a flexible tip scope, further reducing post-operative fever and improving SFR. Direct in-scope suction (DISS) allows synchronous irrigation and suction via dual lumen designs, facilitating the efficient removal of larger stones. Despite encouraging outcomes, suction systems introduce additional procedural complexities. There remains a lack of standardised protocols and robust randomised controlled trials (RCTs) directly comparing different suction systems. Further multi-centre RCTs are warranted to establish best practice clinical guidelines for the safe and effective use of suction in modern endourology.

## Introduction and background

Kidney stone disease is a common urological condition, affecting at least one in 10 people during their lifetime. Around 12% of men and 6% of women will experience at least one episode of renal colic, representing a significant burden to the National Health Service (NHS) [1]. The increasing incidence of urolithiasis has led to rapid technological advances in endourology, including the use of single-use flexible ureteroscopes (F-URS), high-efficiency laser fibres and techniques.

Kidney stones can consist of various compositions, with calcium oxalate and calcium phosphate being the most common. Other types include uric acid, struvite and cystine stones. The risk factors for stone formation include inadequate fluid intake, high protein and salt consumption and metabolic disorders such as obesity, diabetes and hypertension. Additionally, procedures that may cause malabsorption, such as Roux-en-Y gastric bypass and sleeve gastrectomy, can also contribute to the formation of kidney stones. Stones form due to supersaturation of urine with stone-forming material, which leads to crystallisation within the renal parenchyma. These crystals coalesce and continue to grow over time. Two main theories describe this process: the free particle and fixed particle concepts. Free particle theory suggests that the crystal aggregates and grow within the renal tubules, causing urinary obstruction. The fixed particle concept states that stones form through attachment to subepithelial calcific plaques, known as Randall’s plaques. These plaques are deep within the basement membrane of the loop of Henle [2].

The European Association of Urology (EAU) guidelines recommend that stones up to 2 cm can be treated with retrograde intrarenal surgery (RIRS). Despite growing techniques and technology, RIRS still has complications such as residual fragments and infections, including life-threatening sepsis. Stone-free rates (SFRs) are closely linked to surgical expertise and appropriate surgical techniques. Stone fragments of 2-4 mm can cause recurrence or re-presentation, often necessitating additional procedures and increasing operative time. Whilst suction technology has been used for over 25 years in percutaneous nephrolithotomy (PCNL), its integration into ureteroscopy (URS) is relatively new. The application of suction during URS aims to improve fragment clearance, visibility and irrigation control, whilst potentially reducing intrarenal pressure and post-operative complications. The advancement in technology has led to the development of urethral access sheaths (UAS), leading to the option of suction being used in rigid and later in flexible URS (F-URS) [3]. Since 2022, newer scopes have an additional suction channel within the scope itself, such as the use of direct in-scope suction (DISS) [4]. This review focuses on the three main suction approaches: suction urethral access sheaths (SUAS), flexible and navigation suction access sheaths (FANS) and DISS. The aim of the paper is to evaluate the safety, efficacy and clinical outcomes of suction-assisted URS in the management of kidney stones.

Methodology

A literature search was conducted in PubMed and Google Scholar for studies published between January 2015 and October 2025 using combinations of the keywords: ‘suction’, ‘ureteroscopy’, ‘URS’, ‘F-URS’, ‘kidney stones’, ‘FANS’, ‘flexible and navigation suction sheaths’, ‘suction urethral access sheaths’, ‘SUAS’, ‘direct-in-scope suction’, ‘DISS’ and ‘ureterorenoscopy’. References from articles were also reviewed to ensure inclusion of additional relevant evidence. All study designs were considered for this narrative review; however, systematic review and meta-analysis were preferred. Case reports or papers with a non-suction or antegrade approach to ureteric/kidney stone surgery were not included. Only English-language papers were included for this review. No formal Preferred Reporting Items for Systematic Reviews and Meta-Analyses (PRISMA) methodology or risk-of-bias assessment was applied due to the narrative design of the review.

## Review

Endourology has revolutionised the management of urinary stone disease, shifting from open to minimally invasive surgery. Over the years, continuous technological development has improved surgical precision and clinical outcomes. Despite the advancement in modern laser technology, stone surgery can still be challenging due to restricted intra-operative visibility caused by dust particles or residual fragments. These limitations may necessitate additional interventions, increasing operative time and overall healthcare costs [5].

The earliest prototypes of UAS were introduced in the 1970s in endoscopic surgery to reduce the need for multiple withdrawals and re-insertion of F-URS into the renal pelvis. Their introduction mitigated the scope buckling within the bladder. This led to the widespread use of UAS in RIRS. EAU has stated that the use of UAS is safe and has encouraged its use in renal stones, especially large or multiple renal stones. This is also reflected in the American Urological Association (AUA) guidelines. Nevertheless, there are risks to the use of UAS, such as increased risk of ureteral wall ischaemic injury, ureteric stricture formation and increased clinical need for post-operative stents. However, this can be mitigated by using an optimal sheath size. This further led to the introduction of suction-assisted sheaths as a valuable adjunct to remove fragments in URS [6]. Suction-assisted scopes allow the direct evacuation of stone fragments through the endoscope channel, thereby reducing visual loss, intrarenal pressure and heat generation. This facilitates more efficient laser time and overall reduction of operative time [5].

Suction urethral access sheaths (SUAS)

SUAS are designed with built-in suction capability, allowing simultaneous residual fragment removal and irrigation. Early literature on SUAS in RIRS did not show many benefits; however, with the introduction of flexible-tip URS, their clinical utility has become increasingly evident [5].

Clinical studies by Zeng et al. [7] and Deng et al. [8] demonstrated that SUAS use improved stone clearance with higher stone clearance rates on post-operative day 1 and day 30. Intra-operative views were reported to be significantly better. Similarly, another observation study reported a high stone clearance of 88% at four weeks and 92.5% at three months, following SUAS [9]. In a comparative study, Zhu et al. evaluated the safety and efficacy of SUAS with traditional ureteral access sheaths (T-UAS). While overall stone-free rates (SFR) were comparable between the groups, the SUAS group showed a significant reduction in operating time along with a reduction in overall complications, compared to T-UAS [10].

Kayano et al. in 2025 conducted a systematic review and meta-analysis on the use of SUAS in URS and RIRS. This systematic review supported that the use of SUAS significantly reduced operation time by 7.98 minutes (p=0.004), making it cost-effective in high-volume clinical settings. There was no significant difference in the duration of hospital stay. Complications include fever, septic shock and haematuria; however, all complications were significantly reduced with the use of SUAS (risk ratio (RR): 0.49, p<0.001), particularly fever (RR: 0.37, p<0.001). There was no significant difference in post-operative septic shock or haematuria. SFR was significantly improved by 23% in the SUAS group, which may still continue to reduce post-operative stone-related burden [11]. Despite these advantages, SUAS was limited due to its rigidity, which led to the introduction of suction in flexible-tip URS to overcome these anatomical limitations.

Flexible and navigation suction sheaths (FANS)

Flexible and navigation suction sheaths (FANS) incorporate an integrated suction mechanism within a flexible-tip scope, allowing for continuous aspiration of irrigation fluid. As per AUA guidelines, F-URS remains the gold standard treatment for renal stones that are less than 2 cm in size. Recently, with the development of suction techniques, FANS are on the market for use in renal endoscopic surgery [12].

Liu et al. conducted a meta-analysis on the efficacy and safety of FANS compared to T-UAS. The use of FANS had a significantly higher SFR compared to T-UAS. While operative time and hospital stay were comparable between both groups, FANS demonstrated lower complication rates, particularly in reducing post-operative fever and infection risk through improved control of intrarenal pressure, pyelovenous backflow and bacterial translocation [13]. These findings were further supported by a large multi-centre study, which reported consistently high stone clearance rates with FANS and minimal serious adverse events or re-intervention requirements, reinforcing its safety and efficacy as a valuable adjunct in modern endourological practice [14].

A systematic review by Uy et al. in 2025 compared the use of FANS against T-UAS. SFR was significantly better in the FANS group compared to T-UAS (RR: 1.15, p<0.001). However, the review highlighted that there was no significant difference in the zero-fragment rate (ZFR). There was a significant reduction in the re-treatment rates in the FANS in comparison to the T-UAS (RR: 0.34, p=0.006). FANS were associated with lower complication rates, mainly lower infectious rates (RR: 0.45, p<0.001) and urosepsis (RR: 0.43, p=0.005). This is attributed to lower intrarenal pressure, secondary to concurrent suction and lower pyelotubular backflow. No significant difference was noted in the operative time. The optimum sheath size is still under debate, with some surgeons preferring a smaller diameter due to better success at access to the renal pelvis. Although this may result in reduced irrigant flow, with also difficulty in removing larger fragments [15]. This led to the development of dual-channel scopes, which allow for better integration of suction and irrigation fluid in URS, as seen in DISS.

Direct in-scope suction (DISS)

Direct in-scope suction (DISS) URS are single- or dual-channel designs. With a single channel, suction must alternate with irrigation. Alternatively, a dual channel allows for synchronous irrigation and suction. Early studies using single-lumen URS reported an initial SFR of 84%. The combination of DISS with ureteral access sheath (UAS) improved stone clearance from 82% to 97%, suggesting that intermittent in-scope suction enhances stone clearance. The improved dual lumen allows for continuous flow, showing 94% stone clearance, compared to 65% in single-lumen URS [16,17].

Yildirim et al. in 2025 did a retrospective analysis comparing FANS to DISS and showed that the mean operative time was significantly shorter in the FANS group compared to the DISS group (71.8 minutes versus 79.1 minutes). SFR was higher in the FANS group compared to the DISS group (62.5% versus 46.4%). Complete stone clearance was also higher in the FANS group (43.7% versus 32.1%). Nevertheless, the DISS approach was proven to be more cost-effective due to a reduction in the use of access sheaths [18]. Comparing DISS to T-URS, Geavlete et al. found no statistically significant difference in the operative time between the two groups, with no significant operating time difference between single- and dual-lumen URS [19]. This was also supported in the study by Matlaga et al. in 2024, which highlighted that there was no significant difference between T-URS and DISS, but the DISS group had fewer secondary procedures [20]. Studies reporting a longer operative time in DISS highlighted that stones were much larger in the DISS group, comparatively. Overall, DISS improved visibility by clearing blood and reducing laser injury, intrarenal pressure and infective risk [21].

Overall, a systematic review and meta-analysis conducted comparing suction versus T-URS demonstrated that SFR was superior by 12%, but infection complications such as sepsis, infection and fever were lower in the suction group by 56%-76% [3]. Table 1 summarises key studies evaluating various suction-assisted URS and their clinical outcomes.

**Table 1:** Summary of key studies evaluating suction-assisted URS (SUAS, FANS and DISS) URS: ureteroscopy, SUAS: suction urethral access sheath, FANS: flexible and navigation suction sheaths, DISS: direct in-scope suction, OR: odds ratio, Fr: French, SIRS: systemic inflammatory response syndrome, T-UAS: traditional ureteral access sheath, SFR: stone-free rate Data adapted from [7,10,14,18-24]

Key studies	Year of study	Type of study	Technique of suction	Results
Zeng et al. [7]	2016	Clinical observation	SUAS versus T-UAS	SFR is 97% immediately and 100 % at one-month follow-up for SUAS.
Zhu et al. [10]	2018	Matched-pair analysis	SUAS versus T-UAS	Higher SFR (82.4% and 71.5%; SUAS and T-UAS comparatively). Reduction in the operative time in the SUAS group. No difference in the rates of septic shock, haematuria, steinstrasse and urethral stricture. Overall complications are significantly higher in the T-UAS.
Qian et al. [22]	2022	Matched-pair analysis	SUAS versus T-UAS	SFR was significantly higher in SUAS than T-UAS (86.4% versus 71.6%). Lower rates of post-operative fever and SIRS in the SUAS group compared to the T-UAS group (3.7% versus 14.85 and 1.23% versus 12.35).
Gauhar et al. [23]	2025	Multi-centre prospective study	FANS	Bilateral zero residual fragment achievable (42.6%). No recorded sepsis or blood transfusion; 1.7% reported grade 1 ureteric injury.
Yildirim et al. [18]	2025	Retrospective analysis	FANS versus DISS	The mean operative time was significantly shorter in the FANS group versus the DISS group (71.8 minutes versus 79.1 minutes). SFR was higher in the FANS group compared to the DISS group (62.5% versus 46.4%). Complete stone clearance was higher in the FANS group (43.7% versus 32.1%). Post-operative fever rates and hospitalisation duration were similar in both groups.
Liu et al. [13]	2025	Systematic review	FANS versus T-UAS	Higher stone clearance in FANS at one- and 30-day follow-up (OR: 4.01 and 2.37, respectively). Lower rates of post-operative fever (OR: 0.31).
Kwok et al. [24]	2024	Multi-centre outcome analysis	FANS (10/12 Fr sheath) versus FANS (11/13 or 12/14 Fr sheath)	Total operative time was longer in the smaller sheath group (35 minutes versus 32 minutes). No significant difference in SFR. No difference in post-operative complication rates or sepsis.
Geavlete et al. [19]	2024	Prospective comparative study	DISS versus T-UAS	SFR of 97% for DISS with UAS compared to 82.9% with conventional T-UAS.
Matlaga et al. [20]	2024	Prospective randomised study	DISS versus standard URS	No significant differences. However, the DISS group had fewer secondary procedures. No differences in complications.

Discussion

The use of suction-assisted URS represents an important innovation in RIRS. There are limitations to this narrative review due to the limited randomised controlled trials (RCTs). The available data are largely derived from a range of observational studies to systematic reviews rather than RCTs, which may reduce the strength of conclusions due to heterogeneity across study designs, suction systems, stone sizes and patient characteristics. Despite these limitations, studies have supported the use of suction as an effective mechanism by reducing intrarenal pressure and pyelolymphatic/pyelovenous reflux. Suctioning can also improve intra-operative visualisation by removing debris, blood and residual fragments simultaneously [5].

Both SUAS and FANS have been shown to have better stone clearance, with a reduction in post-operative fever and SIRS response. SUAS have a significant reduction in operating time, whilst FANS have shown promising results in bilateral URS with zero residual fragments in one operation setting [7,10,14,18,22-24]. DISS provides a more cost-effective option, due to the lack of need for an access sheath, whilst also being effective at stone clearance [18]. However, suction devices can introduce additional procedural complexities due to the need for additional tubing and control mechanism. At present, there are no standardised suction mechanisms or guidelines, causing a huge variation in practice. The use of suction also requires a higher volume of irrigation fluid to prevent pelvicalyceal collapse [5]. Given these complexities and lack of standardised guidance, multi-centre RCTs are required to compare the different suction technologies in their use in RIRS to establish standardised clinical protocols to guide their safe and effective use.

Despite limited RCTS available, a recent propensity-matched pair analysis was performed, looking at data from two global registries: the suction mini-PCNL registry (STUMPS) and the FANS in URS registry. The FANS registry included 686 patients across 25 centres in 20 countries. FANS demonstrated significant recovery time with a reduction in post-operative pain and need for re-intervention. Whilst suction in URS proves to be safe and efficient, it is important to acknowledge that this study was not a direct comparison between all suction devices in retrograde URS but was a comparison study against suction in PCNL [25].

## Conclusions

Suction-assisted URS is an evolving field, with multiple innovative devices demonstrating potential benefits in improving stone clearance rates and reducing overall complications such as post-operative fever and infections. Each suction system (SUAS, FANS and DISS) has its own advantages and limitations. However, lack of standardized protocols, guidelines and robust comparative studies limits widespread adoption. Further multi-centre RCTs are required to compare available suction systems to optimise safe use in RIRS. Establishment of standardized guidance is also very important to ensure safe integration of suction in endourology across different departments.
